# Prevalence of the Peroneus Quartus Muscle and Its Association with Peroneal Tendon Pathologies: An MRI Study of 1160 Ankles

**DOI:** 10.3390/diagnostics15182329

**Published:** 2025-09-14

**Authors:** Yavuz Yuksel, Tarkan Ergun, Ozkan Kose

**Affiliations:** 1 Department of Radiology, Faculty of Medicine, Alaaddin Keykubat University, 07400 Alanya, Turkey; yavuz.yuksel@alanya.edu.tr (Y.Y.); tarkanergun@yahoo.com (T.E.); 2 Department of Orthopedics and Traumatology, Antalya Training and Research Hospital, 07100 Antalya, Turkey

**Keywords:** peroneus quartus, MRI, ankle, peroneal tendon tear, tendinopathy, anatomical variation

## Abstract

**Background/Objectives:** The peroneus quartus (PQ) muscle is a supernumerary muscle in the lateral compartment of the leg. Although frequently asymptomatic, it may contribute to peroneal tendon (PT) disorders due to mechanical crowding in the retromalleolar groove. This study aimed to determine the prevalence of the PQ muscle on MRI and assess its association with PT pathologies. **Materials and Methods:** This retrospective study evaluated 1160 ankle MRI scans from 1073 patients (mean age, 42.7 ± 14.5 years; 643 females, 430 males). The presence of the PQ muscle and associated PT pathologies, including tenosynovitis, tendinitis, and tendon tears of the peroneus brevis tendon (PBT) and peroneus longus tendon (PLT), was recorded. Statistical analyses were performed using chi-square tests, and associations were expressed as odds ratios (OR) with 95% confidence intervals (CI). **Results:** The PQ muscle was identified in 123 of 1160 ankles, corresponding to a prevalence of 10.6%. Its incidence was significantly higher in males (12.7%) than in females (9.2%) (*p* = 0.018), while the side distribution showed no statistically significant difference. PQ presence was associated with PBT pathology, particularly tendinitis and longitudinal tears, and with PLT tendinitis, whereas no clear association was observed with tenosynovitis or PLT tears. **Conclusions:** The PQ muscle is a relatively common anatomical variant, present in 10.6% of ankles on MRI scans. Its presence is significantly associated with PT abnormalities, especially PBT tendinitis and tears. Awareness of PQ may aid clinicians and radiologists in assessing lateral ankle pain and peroneal tendon disorders.

## 1. Introduction

**Figure 1:**
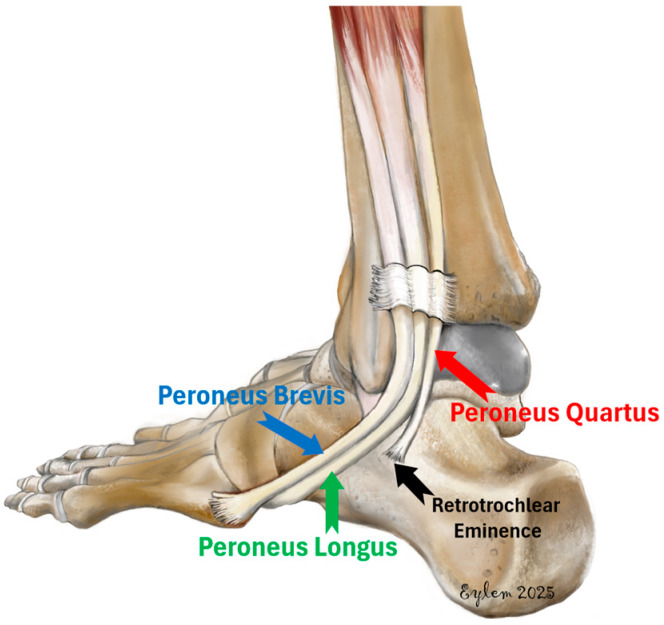
Illustration showing the lateral ankle anatomy with emphasis on the peroneus quartus muscle. The PQ is depicted originating from the peroneus brevis and inserting onto the retrotrochlear eminence of the calcaneus, alongside the normal course of the peroneus longus and brevis tendons.

The peroneus quartus (PQ) muscle, also called fibularis quartus, is a supernumerary muscle of the lateral compartment of the leg, variably present in the human population [1,2]. It is considered a phylogenetic remnant associated with the evolution of bipedal locomotion. Hecker proposed that its presence in humans but absence in other primates represents an adaptive response to bipedal gait, contributing to lateral foot elevation and stabilization of the subtalar joint during pronation and supination [3]. The PQ most commonly originates from the peroneus brevis and inserts on the calcaneus, cuboid, or the lateral tubercle, although anatomical variations have been documented (Figure 1) [4].

While often asymptomatic, the presence of the PQ has been increasingly implicated in lateral ankle pathologies [5,6]. Due to its space-occupying effect in the retromalleolar groove, it may lead to mechanical crowding within the peroneal tunnel, altering the biomechanics of adjacent tendons. As a result, it has been associated with peroneal tendon disorders, including tenosynovitis, tendon tears, and tendon subluxation, especially in symptomatic individuals [7,8].

With the widespread use of magnetic resonance imaging (MRI) in ankle evaluations, the incidental identification of the PQ has become more frequent. However, its clinical relevance remains a matter of debate. Some authors consider it a benign anatomical variant [9,10], whereas others have reported a significant association between the PQ and degenerative or inflammatory changes in the peroneal tendons [5,7,8]. Despite its potential role in peroneal tendon pathology, comprehensive epidemiological data from large magnetic resonance imaging (MRI)-based cohorts remain limited [6]. Furthermore, differences, in PQ prevalence concerning sex and side (laterality) have not been well established, and its relationship with specific peroneal tendon disorders, such as peroneus brevis or longus tendinopathy or tears, is still underexplored. These limitations hinder confident translation of PQ detection into everyday diagnostic reasoning and management.

To address these gaps in the literature, a large MRI-based study of 1160 ankles was conducted, designed to (1) estimate PQ prevalence and its sex- and side-specific distribution; (2) describe bilateral occurrence patterns; and (3) evaluate the association between PQ and specific peroneal tendon pathologies (PBT/PLT tendinitis and tears and tenosynovitis) using predefined MRI criteria. We hypothesized that the presence of the PQ muscle would be significantly associated with an increased frequency of peroneal tendon disorders, particularly tendinitis and tendon tears, due to its mechanical interference and space-occupying effect within the peroneal tunnel.

## 2. Materials and Methods

### 2.1. Patients and Study Design

This retrospective cross-sectional study was conducted by reviewing the radiological database of a tertiary university hospital. Ankle magnetic resonance imaging (MRI) scans obtained between June 2021 and October 2023 were evaluated. All patients who underwent ankle MRI for any clinical indication were assessed for eligibility. Exclusion criteria included patients with postoperative changes, traumatic gross edema, infection, tumoral lesions, inflammatory joint diseases, or congenital foot deformities observed on MRI. In addition, examinations were excluded if MRI scans were of inadequate quality for evaluation due to technical or image artifacts caused by scanner malfunction or patient-related factors or if the lateral ankle compartment was not fully visualized. Demographic data, including patient age, sex, and laterality of the examined ankle, were recorded. The study protocol was approved by the Institutional Clinical Research Ethics Committee of Alanya University, Training and Research Hospital (Approval Date and No: 21.05.2025/09-15) and was conducted in accordance with the principles of the Declaration of Helsinki.

### 2.2. Magnetic Resonance Imaging Protocol

MRI examinations were performed using two 1.5 Tesla scanners: GE Signa Explorer (GE Healthcare, Chicago, IL, USA) and Siemens Magnetom Altea (Siemens Healthineers, Erlangen, Germany). All patients were imaged in the supine position with the ankle positioned neutrally. A 16-channel coil was used for the GE system, and a 20-channel coil was used for the Siemens system. The protocol comprised sagittal, axial, and coronal planes. Sequence families and their parameter ranges (TR/TE, slice thickness, and field of view and acquisition time) are summarized in Table 1. The typical scan time for the complete ankle protocol was approximately 15–20 min per ankle, varying with patient factors and the need for repeat localizers. The image review was performed on the institution’s Picture Archiving and Communication System (PACS).

### 2.3. Assessment of the Peroneus Quartus Muscle and Peroneal Pathologies

**Figure 2:**
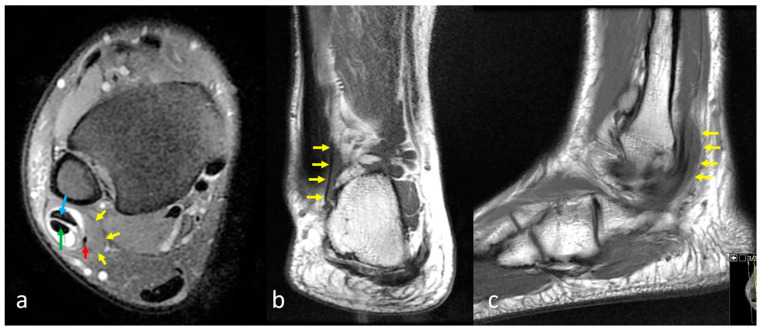
MRI appearance of the peroneus quartus (PQ) muscle in the lateral compartment of the leg. (**a**) Axial fat-suppressed proton density–weighted fast spin echo (FSE) image showing the peroneus brevis (blue arrow), peroneus longus (green arrow), and the accessory peroneus quartus muscle (red arrow). The PQ lies posterior to the fibula and lateral to the other peroneal tendons. Yellow arrows indicate the muscle belly of the PQ. (**b**) Coronal T1-weighted FSE image demonstrating the longitudinal course of the PQ muscle (yellow arrows) descending toward the calcaneus. (**c**) Sagittal T1-weighted fast spin echo (FSE) image demonstrating the peroneus quartus (PQ) muscle extending distally toward its insertion on the retrotrochlear eminence of the calcaneus (yellow arrows).

The presence or absence of the peroneus quartus (PQ) muscle was assessed on axial images in the retromalleolar region. PQ was defined as an accessory muscle-tendon unit coursing posterior or medial to the peroneus brevis and longus tendons, typically inserting onto the calcaneus (Figure 2) [11]. All MRI scans were jointly reviewed by two radiologists with more than ten years of experience in musculoskeletal MRI. Evaluations were performed in consensus, and the presence of the PQ muscle and peroneal tendon pathologies was recorded.

### 2.4. Definitions of Peroneal Tendon Pathologies

**Figure 3:**
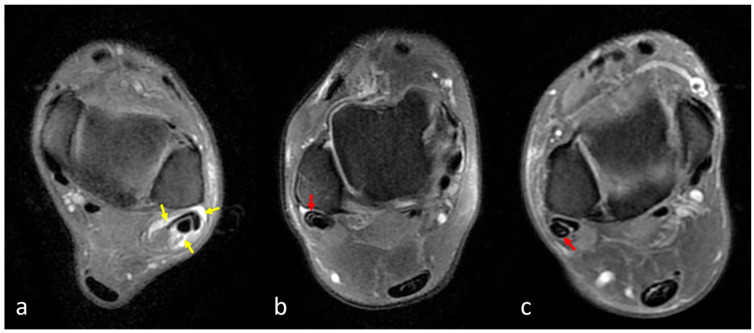
(**a**) Axial fat-suppressed proton density–weighted fast spin echo (FSE) MRI demonstrating peroneal tenosynovitis. A high-signal-intensity rim is observed surrounding the peroneal tendons (yellow arrows), indicating abnormal fluid accumulation within the tendon sheath. The fluid thickness exceeds 3 mm at its widest point, consistent with the diagnosis of tenosynovitis. (**b**,**c**) Axial fat-suppressed proton density–weighted fast spin echo (FSE) MR images demonstrating peroneal tendinopathy. Increased intratendinous signal intensity of the peroneus brevis (red arrow) (**b**) and longus tendons (**c**) (red arrow) is seen without fiber discontinuity. The findings are consistent with peroneal tendinopathy (tendinitis).

**Figure 4:**
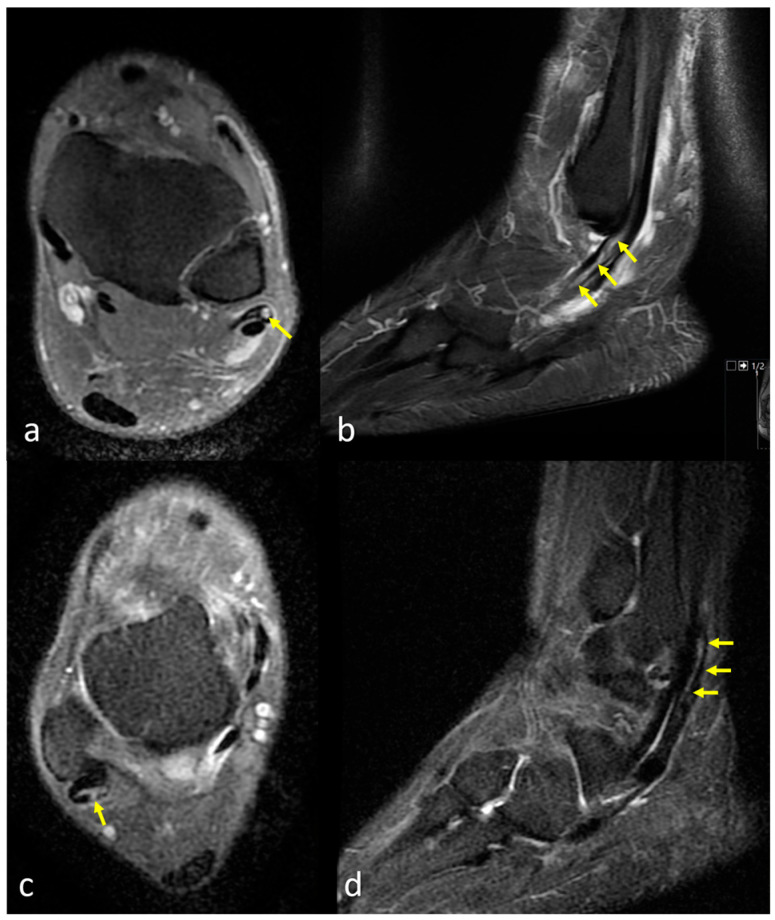
(**a**) Axial fat-suppressed proton density–weighted fast spin echo (FSE) image shows discontinuity and intratendinous fluid signal within the PB tendon (yellow arrow), consistent with a tendon tear. (**b**) Sagittal short tau inversion recovery (STIR) image reveals longitudinal splitting and abnormal morphology of the PB tendon (yellow arrows), further confirming the diagnosis of tendon tear. (**c**) Axial fat-suppressed proton density–weighted fast spin echo (FSE) image shows increased signal intensity and structural irregularity within the PL tendon (yellow arrow), indicating a tendon tear. (**d**) Sagittal short tau inversion recovery (STIR) image reveals discontinuity and intratendinous hyperintensity along the course of the PL tendon (yellow arrows), consistent with a longitudinal tear.

***Tenosynovitis:*** Tenosynovitis was defined as abnormal fluid accumulation surrounding an otherwise intact tendon within the synovial sheath and was considered present when the fluid exceeded 3 mm at its widest point (Figure 3a) [12]. ***Peroneal Tendinopathy (Tendinitis):*** Peroneal tendinopathy (tendinitis) was defined as irregular tendon contours and/or intratendinous intermediate signal increase on fluid-sensitive sequences (Figure 3b,c). ***Peroneal Tendon Tear:*** Peroneal tendon tear was considered present in cases demonstrating partial or complete fiber discontinuity and/or focal areas of high signal intensity within the tendon on fluid-sensitive sequences (Figure 4). Tendinopathy and tear evaluations were made separately for the peroneus brevis tendon (PBT) and peroneus longus tendon (PLT).

### 2.5. Statistical Analysis

Descriptive statistics were used to summarize patient demographics and prevalence data and are presented as means with standard deviations (SD) for continuous variables and as frequencies with percentages for categorical variables. Categorical variables were compared using the chi-square test or Fisher’s exact test, as appropriate. Logistic regression analysis was conducted to assess the association between the presence of the peroneus quartus (PQ) muscle and peroneal tendon pathologies, with results reported as odds ratios (OR) and 95% confidence intervals (CI). A *p*-value of <0.05 was considered statistically significant. Statistical analyses were performed using IBM SPSS Statistics for Windows, Version 23.0 (IBM Corp., Armonk, NY, USA).

## 3. Results

A total of 1073 patients were included in the study, with a mean age of 42.7 ± 14.5 years. Of these, 643 were female (mean age: 45.5 ± 14.0 years) and 430 were male (mean age: 38.4 ± 14.2 years). Male patients were significantly younger than female patients (*p* < 0.001). Bilateral ankle MRI examinations were available for 87 patients (53 females, 34 males), resulting in a total of 1160 ankle MRI scans for analysis. Among these, 594 (51.2%) were left-sided and 566 (48.8%) were right-sided.

The PQ muscle was identified in 123 out of 1160 ankle MRIs, yielding an overall prevalence of 10.6%. The detection rate was significantly higher in male ankles (12.7%) compared to female ankles (9.2%) (*p* = 0.018). The PQ was more frequently located on the right side (60.2%) than the left side (39.8%), although this difference did not reach statistical significance (*p* = 0.064) (Table 2).

Among the 87 patients with bilateral ankle imaging, the PQ muscle was absent bilaterally in 70 patients (80.5%), unilaterally present in 12 patients (13.8%), and bilaterally present in 5 patients (5.7%). There was no significant difference in distribution between males and females (*p* = 0.579) (Table 3).

The presence of the PQ muscle was evaluated in relation to several peroneal tendon pathologies. Statistical analysis revealed a significant association between the presence of PQ and peroneus brevis tendon (PBT) tendinitis (OR = 3.06; 95% CI: 1.73–5.41; *p* = 0.001), PBT tear (OR = 3.64; 95% CI: 1.64–8.10; *p* = 0.003) and peroneus longus tendon (PLT) tendinitis (OR = 2.43; 95% CI: 1.56–3.79; *p* = 0.001). In contrast, no statistically significant association was found between the presence of PQ and tenosynovitis (OR = 1.47; 95% CI: 0.56–3.88; *p* = 0.396) or PLT tear (OR = 1.05; 95% CI: 0.06–19.97; *p* = 0.638) (Table 4).

## 4. Discussion

In this retrospective MRI-based study evaluating 1160 ankles, the peroneus quartus (PQ) muscle was identified in 10.6% of cases. The incidence was significantly higher in males (12.7%) compared to females (9.2%). Among the 87 patients with bilateral ankle MRIs, the PQ muscle was found bilaterally in 5.7% of cases. Additionally, a statistically significant association was observed between the presence of the PQ muscle and specific peroneal tendon pathologies, namely peroneus brevis tendon (PBT) tendinitis and tear, as well as peroneus longus tendon (PLT) tendinitis. No significant relationship was found between PQ and tenosynovitis or PLT tear. These findings support the hypothesis that the PQ muscle, due to its space-occupying effect in the retromalleolar groove, contributes to mechanical crowding and subsequent degenerative or inflammatory changes in adjacent tendons.

The reported prevalence of the peroneus quartus (PQ) muscle in the literature exhibits considerable variation, influenced by the characteristics of the study population, sample size, geographical factors, and, particularly, the method of detection, whether through imaging techniques or cadaveric dissection (Table 5) [2,3,4,6,7,8,9,10,11,13,14,15,16,17,18,19,20,21,22,23,24,25,26,27,28,29,30,31,32,33,34,35,36,37,38,39,40,41,42,43,44,45,46,47]. Prevalence estimates range widely, from as low as 0.98% [29] to as high as 55.6% [17], without a clear geographical pattern. Significant variation is evident even among studies conducted within the same country and between neighboring countries sharing similar ethnic backgrounds [11,39]. Detection methods further contribute to this variability, with cadaveric studies generally reporting higher prevalence rates, likely due to direct visualization and meticulous anatomical dissection, allowing for more precise identification of smaller or rudimentary muscle variations [34,36]. In contrast, imaging-based studies tend to report somewhat lower prevalences, possibly reflecting limitations in sensitivity and specificity compared to direct anatomical examination [11,35]. Notably, the current study represents the most extensive patient series reported to date, evaluating 1073 patients (1160 ankles) through MRI and revealing a prevalence of 10.6%, closely aligning with the cumulative prevalence of 11.3% derived from a total of 6236 ankles across all reviewed studies. Overall, these findings emphasize the complex interplay between methodology, population characteristics, and detection techniques in determining the prevalence of the PQ muscle [48].

Sex-based differences in the prevalence of the PQ muscle have been inconsistently reported, and many studies do not provide a sex-specific breakdown of their findings. However, among the studies that report such data, a trend toward a slightly higher prevalence appears to be present in males. In the current MRI-based study, PQ was more frequently observed in male patients (12.7%) than in females (9.2%), with a statistically significant difference (*p* = 0.018). Similarly, Hur et al. reported PQ in 9 male and 4 female cadavers among 40 individuals (38 males and 42 females), indicating a higher occurrence in males (11.2% vs. 5%) despite the female-dominant cadaver pool [39]. In the classic anatomical study by Sobel et al., 27 PQ muscles were found in 124 cadaveric legs (21.7% overall), with 15 (12.1%) occurring in males and only 5 in females (9.6%), further supporting the male predominance [4]. In contrast, the study by Nascimento et al. found the PQ in 4.3% of males and 3.3% of females in their MRI-based sample of 211 patients, though the difference was not statistically significant [35]. In the study by Pota et al., the PQ muscle was identified in 117 out of 849 ankles (13.8%). When broken down by sex, it was found in 14.5% of male ankles and 13.4% of female ankles. The difference between sexes was not statistically significant (*p* = 0.470), indicating that the prevalence of the PQ muscle did not differ meaningfully between men and women [6]. Despite this trend, the absence of sex-specific data in a substantial number of studies limits the ability to draw definitive conclusions. Nonetheless, the consistent pattern of higher PQ detection rates in males across multiple cadaveric and imaging-based studies suggests a genuine sex-linked anatomical variation that warrants further investigation.

In our study, the presence of the PQ muscle was significantly associated with peroneus brevis tendon pathology, particularly tendinitis and longitudinal tears, while no significant association was observed with peroneus longus tendon tears or tenosynovitis. These findings support the notion that the PQ muscle may contribute to selective mechanical stress and degeneration of the peroneus brevis tendon. These findings underscore the potential clinical relevance of the PQ muscle as a predisposing anatomical variant in lateral ankle disorders. Our results are consistent with previous reports suggesting that the PQ muscle may contribute to mechanical crowding within the retromalleolar groove, increasing the risk of tendon pathology. For instance, Galli et al. reported that in 12.2% of their sample population, the presence of PQ was associated with pain or peroneal tendon tears, emphasizing its potential clinical implications [40]. Furthermore, Rosenberg et al. provided radiological evidence that the PQ muscle, alongside other anatomical variants, such as a convex retromalleolar groove and low-lying muscle bellies, was frequently observed in patients with longitudinal splits of the peroneus brevis tendon. In their study, 2 of the 27 patients with surgically confirmed tendon tears had a PQ muscle, suggesting a pathophysiological link [7]. Other authors have also echoed this correlation between the presence of PQ and peroneal tendon injuries. For instance, Lamm et al. [8] and Miura et al. [9] highlighted the importance of identifying accessory peroneal muscles in patients presenting with lateral ankle pain and mechanical symptoms, as surgical excision led to symptom resolution in selected cases. Similarly, Ayanoglu et al. [5] demonstrated that PQ was more frequently detected in patients with peroneal tendon disorders compared to the general population. Taken together, the literature supports our observation that PQ is not merely an incidental anatomical variation but may have important clinical consequences. However, the pathogenic potential of the PQ may be influenced by factors such as muscle size, tendon anatomy, and mechanical demand, which were not directly measured in most studies.

Recognition of the PQ muscle and its association with peroneal tendon pathology is crucial, particularly in patients with chronic lateral ankle pain or instability. MRI remains the most reliable tool for detecting and evaluating associated tendon abnormalities [7]. An efficient MRI approach is to review axial fat-suppressed PD/FSE images at the retromalleolar groove for an accessory structure coursing posteromedial to the peroneus brevis/longus, confirm longitudinal continuity on coronal T1/PD-FS images, and delineate the muscle belly and distal insertion on sagittal planes. Ultrasonography can also identify PQ and peroneal tendon pathologies; moreover, it can dynamically assess tendon subluxation at the retromalleolar groove, although it is operator-dependent [25].

PQ on MRI should not be interpreted as pathologic per se; rather, it should refocus evaluation on symptom–lesion concordance. In cases of lateral ankle pain, snapping, or peroneal weakness, the identification of PQ should prompt a targeted search for associated peroneal tendon pathology. Such pathology may manifest as peroneus brevis longitudinal splits, peroneus longus tendinopathy, and tenosynovitis, in addition to signs of mechanical crowding within the peroneal tunnel. Examples of such crowding may be an accessory muscle–tendon unit sharing the retromalleolar groove, tendon flattening/impingement, or sheath fluid. Treatment planning is individualized based on symptomatology and coexisting lesions [49,50,51]. Incidental/asymptomatic PQ requires no treatment. Mild symptomatic cases without tendon tears are typically managed conservatively, including activity modification, short-term immobilization as needed, peroneal strengthening and proprioception exercises, footwear/orthosis optimization, and image-guided peroneal sheath injections. Persistent symptoms with imaging evidence of crowding and/or tendon lesions warrant consideration of tendoscopic or open PQ debulking/excision, with concomitant treatment of peroneal pathologies and stabilization procedures when indicated [49].

## 5. Strengths, Limitations, and Future Directions

A major strength of this study lies in its large sample size and standardized assessment of peroneal tendon pathologies by radiologists experienced in musculoskeletal radiology. To the best of our knowledge, this is one of the largest MRI-based series evaluating both the prevalence and clinical impact of the PQ muscle. However, several limitations should be acknowledged. First, the retrospective nature of the study limits causal inference, and clinical symptoms of the patients were not correlated with imaging findings. Second, variability in MRI acquisition protocols and magnet strengths may have influenced muscle detection. Finally, the lack of histological or intraoperative confirmation limits the definitive identification of PQ in ambiguous cases. Additionally, while MRI is a powerful diagnostic tool, subtle anatomical variants may be missed or misinterpreted. Future studies should aim to prospectively investigate the clinical significance of the PQ muscle in symptomatic patients, with standardized imaging protocols and correlation to surgical or histological findings. Longitudinal studies would also be valuable for clarifying whether the presence of PQ predisposes individuals to progressive tendon degeneration or symptomatic lateral ankle instability.

## 6. Conclusions

In conclusion, the PQ muscle is a relatively common anatomical variant, identified in over 10% of ankle MRIs in our cohort. Its presence was significantly associated with tendinopathic changes and tears of the peroneus brevis and longus tendons, supporting the notion that it may have clinical relevance in the context of lateral ankle pain or dysfunction. Awareness of this muscle and its potential effects on tendon integrity may aid radiologists and clinicians in the evaluation and management of peroneal tendon disorders.

## Figures and Tables

**Table 1:** Ankle MRI acquisition parameters. Abbreviations: STIR: short tau inversion recovery, TR: repetition time, TE: echo time, FOV: field of view, TSE: turbo spin echo, FSE: fast spin echo.

Plane	Sequence	TR (ms)	TE (ms)	Slice (mm)	FOV (mm)	Acquisition Time
**Sagittal**	STIR	1800–5500	9.1–136.3	4.0	160	216 s
	T1-w FSE	300–650	12.6–50.6	4.0	160	134 s
	T2-w TSE (with STIR)	2500–6000	10–61	3.5	200	122 s
	T1-w TSE	350–650	10–30	3.5	200	50 s
**Axial**	PD-w FSE, fat-suppressed	1500–3000	8.5–76.9	5.0	160	113 s
	PD-w TSE, fat-suppressed	3200–4500	10–82	4.0	180	115 s
**Coronal**	T1-w FSE	300–1500	12.6–50.6	4.0	160	122 s
	T1-w TSE	537–650	11–32	4.0	180	102 s
	PD-w, fat-suppressed	152–2500	12.6–113.8	4.0	160	135 s
	PD-w TSE, fat-suppressed	3300–4000	10–83	4.0	180	131 s

**Table 2:** Distribution of the peroneus quartus muscle according to sex and laterality.

Gender	PQ Present	PQ Absent	*p*-Value
***Male (n, % within males)***	59 (12.7%)	405 (87.3%)	0.064 ^1^
***Female (n, % within females)***	64 (9.2%)	632 (90.8%)	
***Total (n, % within total)***	123 (10.6%)	1037 (89.4%)	
	**Right**	**Left**	***p*-value**
***Male (n, % within males)***	38 (64.4%)	21 (35.6%)	0.150 ^1^
***Female (n, % within females)***	36 (56.2%)	28 (43.8%)	
***Total (n, % within total)***	74 (60.2%)	49 (39.8%)	

^1^ Chi-square test. Values are given as n (row %), and percentages sum to 100% within each row.

**Table 3:** Frequency of unilateral and bilateral presence of the peroneus quartus muscle stratified by sex.

Peroneus Quartus	Overall	Male	Female	*p*-Value
**Bilateral Absent**	70 (80.5%)	27 (79.4%)	43 (81.1%)	0.579
**Unilateral Present**	12 (13.8%)	4 (11.8%)	8 (15.1%)	
**Bilateral Present**	5 (5.7%)	3 (8.8%)	2 (3.8%)	
**Total**	87 (100%)	34 (100%)	53 (100%)	

**Table 4:** Association between the presence of the peroneus quartus muscle and peroneal tendon pathologies. Abbreviations: PBT: peroneus brevis tendon, PLT: peroneus longus tendon, CI: confidence interval.

Pathologies		Peroneus Quartus		*p*-Value	Odd’s Ratio (95% CI)
		Absent	Present		
**Tenosynovitis**	***No***	1008 (89.5%)	118 (10.5%)	0.396	1.47 (0.56–3.88)
	***Yes***	29 (85.3%)	5 (14.7%)		
**PBT Tendinitis**	***No***	982 (90.3%)	105 (9.7%)	0.001	3.06 (1.73–5.41)
	***Yes***	55 (75.3%)	18 (24.7%)		
**PBT Tear**	***No***	1015 (89.9%)	114 (10.1%)	0.003	3.64 (1.64–8.1)
	***Yes***	22 (71.0%)	9 (29.0%)		
**PLT Tendinitis**	***No***	906 (90.9%)	91 (9.1%)	0.001	2.43 (1.56–3.79)
	***Yes***	131 (80.4%)	32 (19.6%)		
**PLT Tear**	***No***	1033 (89.4%)	123 (10.6%)	0.638	1.05 (0.06–19.97)
	***Yes***	4 (100%)	0 (0%)		

Fisher’s Exact test. Values are given as n (row %), and percentages sum to 100% within each row.

**Table 5:** List of previous studies that report the prevalence of PQ muscle in the current literature.

Author	Year	Country	Study Type	Number of Patients (Ankles) *	Overall Prevalence
Wood [13]	1868	UK	Cadaver	140 ankles	1.4% (2/140)
Pozzi [14]	1872	France	Cadaver	52 ankles	7.7% (4/52)
Macalister et al. [15]	1875	UK	Cadaver	60 ankles	13.3% (8/60)
Gruber [16]	1879	Russian	Cadaver	982 ankles	12% (118/982)
Testut [17]	1884	France	Cadaver	36 ankles	55.6% (20/36)
Chudzinski [18]	1898	France	Cadaver	30 ankles	23.3% (7/30)
Hecker [3]	1923	France	Cadaver	47 ankles	13% (6/47)
Pales et Chippaux [19]	1952	Vietnam	Cadaver	36 ankles	19.4% (7/36)
Mori [20]	1964	Japan	Cadaver	73 ankles	2.7% (2/73)
Sobel et al. [4]	1990	USA	Cadaver	65 (124 ankles)	21.7% (27/124)
Chaney et al. [21]	1996	USA	Cadaver	269 ankles	3% (8/269)
Rosenberg et al. [7]	1997	USA	MRI	37 (41 ankles)	4.9% (2/41)
Cheung et al. [47]	1997	USA	MRI	76 (136 ankles)	10.3% (14/136)
Bonnin et al. [22]	1997	France	Surgical	18 (18 ankles)	16.7% (3/18)
Kudoh et al. [23]	1999	Japan	Cadaver	24 ankles	12.5% (3/24)
Major et al. [10]	2000	USA	MRI	42 ankles	23.8% (10/42)
DiGiovanni et al. [24]	2000	USA	Surgical	61 (61 ankles)	8.2% (5/61)
Chepuri et al. [25]	2001	USA	USG/MRI	32 ankles	21.8% (7/32)
Borne et al. [26]	2002	France	MRI	63 ankles	11.1% (7/63)
Zammit & Singh [27]	2003	UK	Cadaver	66 (102 ankles)	5.9% (6/102)
Miura et al. [9]	2004	Japan	Cadaver	56 (112 ankles)	5.3% (6/112)
Lamm et al. [8]	2004	USA	Surgical	32 ankles	15.6% (5/32)
Saupe et al. [28]	2007	Switzerland	MRI	65 (65 ankles)	16.9% (11/65)
Tubbs et al. [29]	2008	USA	Cadaver	89 ankles	1.1% (1/89)
Thomas et al. [30]	2009	USA	MRI	7 (7 ankles)	14.2% (1/7)
Ugurlu et al. [31]	2010	Turkey	Cadaver	11 (22 ankles)	9% (2/22)
Park et al. [11]	2010	Korea	MRI	82 ankles	6% (5/82)
Saxena et al. [32]	2011	USA	MRI	100 (102 ankles)	0.98% (1/102)
Prakash et al. [33]	2011	India	Cadaver	70 ankles	4.3% (3/70)
Athavale et al. [34]	2012	India	Cadaver	92 ankles	21.7% (20/92)
Nascimento et al. [35]	2012	Brazil	MRI	135 (211 ankles)	7.6% (16/211)
Clarkson et al. [36]	2013	USA	Cadaver	277 ankles	20.9% (58/277)
Bilgili et al. [37]	2014	Turkey	Cadaver	58 (115 ankles)	5.2% (6/115)
Zhenbo et al. [38]	2014	China	Surgical	26 ankles	15.3% (4/26)
Hur et al. [39]	2015	Korea	Cadaver	40 (80 ankles)	16.3% (13/80)
Galli et al. [40]	2015	USA	MRI	104 (108 ankles)	12.9% (14/108)
M S Somesh et al. [41]	2016	India	Cadaver	47 ankles	4.2% (2/47)
Grace et al. [42]	2016	India	Cadaver	32 (64 ankles)	1.5% (1/64)
Mustafa et al. [43]	2017	Saudi Arabia	Cadaver	20 ankles	20% (4/20)
Dangintawat et al. [44]	2019	Thailand	Cadaver	109 ankles	11.93% (13/109)
Ersoz et al. [45]	2019	Turkey	MRI	60 (69 ankles)	13% (9/69)
Inchai et al. [46]	2021	Thailand	Cadaver	30 (60 ankles)	6.6% (6/60)
Pota et al. [6]	2025	Turkey	MRI	738 (849 ankles)	13.8% (117/849)
Current study	2025	Turkey	MRI	1073 (1160 ankles)	10.6% (123/1160)
Total				6236 ankles	11.3% (708/6236)

Abbreviations: USA: United States of America, UK: United Kingdom, MRI: magnetic resonance imaging, USG: ultrasonography. * In instances where the number of patients and the number of ankles are specified separately, the number of ankles is given in parentheses.

## Data Availability

The datasets are not publicly available. The de-identified data are available upon request to the corresponding author due to privacy, ethical, and legal restrictions protecting patient confidentiality.

## Abbreviations

CI	Confidence Interval
FSE	Fast Spin Echo
MRI	Magnetic Resonance Imaging
OR	Odds Ratio
PD	Proton Density
PBT	Peroneus Brevis Tendon
PLT	Peroneus Longus Tendon
PQ	Peroneus Quartus
STIR	Short Tau Inversion Recovery
TSE	Turbo Spin Echo
TR	Repetition Time
TE	Echo Time
FOV	Field of View

## References

[1] Hallinan J.T.P.D., Wang W., Pathria M.N., Smitaman E., Huang B.K. (2019). The peroneus longus muscle and tendon: A review of its anatomy and pathology. Skelet. Radiol..

[2] Cheung Y. (2017). Normal Variants: Accessory Muscles About the Ankle. Magn. Reson. Imaging Clin. N. Am..

[3] Hecker P. (1923). Study on the peroneus on the tarsus. Anat. Rec..

[4] Sobel M., Levy M.E., Bohne W.H. (1990). Congenital variations of the peroneus quartus muscle: An anatomic study. Foot Ankle.

[5] Ayanoglu T., Arikan E., Kurtbogan M., Yilmaz O.F., Kaya Y.E., Ozturan K.E. (2022). Anatomical Factors Which Influence the Formation of Peroneal Tendon Tears: A Retrospective Comparative Study. J. Foot Ankle Surg..

[6] Pota K., Baysal C.Ç., Civan O., Ürgüden M. (2025). Evaluation of the relations between foot & ankle pathologies and anatomic variations with magnetic resonance imaging of 849 study population. Jt. Dis. Relat. Surg..

[7] Rosenberg Z.S., Beltran J., Cheung Y.Y., Colon E., Herraiz F. (1997). MR features of longitudinal tears of the peroneus brevis tendon. Am. J. Roentgenol..

[8] Lamm B.M., Myers D.T., Dombek M., Mendicino R.W., Catanzariti A.R., Saltrick K. (2004). Magnetic resonance imaging and surgical correlation of peroneus brevis tears. J. Foot Ankle Surg..

[9] Miura K., Ishibashi Y., Tsuda E., Kusumi T., Toh S. (2004). Split lesions of the peroneus brevis tendon in the Japanese population: An anatomic and histologic study of 112 cadaveric ankles. J. Orthop. Sci..

[10] Major N.M., Helms C.A., Fritz R.C., Speer K.P. (2000). The MR imaging appearance of longitudinal split tears of the peroneus brevis tendon. Foot Ankle Int..

[11] Park H.J., Cha S.D., Kim H.S., Chung S.T., Park N.H., Yoo J.H., Park J.H., Kim J.H., Lee T.W., Lee C.H. (2010). Reliability of MRI findings of peroneal tendinopathy in patients with lateral chronic ankle instability. Clin. Orthop. Surg..

[12] Cabral P., Paulino C., Takahashi R., Clopton P., Resnick D. (2013). Correlation of morphologic and pathologic features of the various tendon groups around the ankle: MR imaging investigation. Skelet. Radiol..

[13] Wood J. (1868). Variations in human myology observed during the winter session of 1867–1868 at King’s College, London. Proc. R. Soc. Lond..

[14] Pozzi S. (1872). Sur une varieté fréquente du muscle court péronier latéral chez l’homme (anomalie réversive). Bull. Soc. Anthropol. Paris.

[15] Macalister A. (1875). Additional observations on muscular anomalies in human anatomy (third series), with a catalogue of the principal muscular variations hitherto published. Trans. R. Ir. Acad..

[16] Gruber W. (1879). Ein neuer Musculus peroneo-calcaneus externus anterior. Arch. Pathol. Anat. Physiol. Klin. Med..

[17] Testut L. (1884). Les Anomalies Musculaires Chez l’Homme Expliquées par l’Anatomie Comparée, Leur Importance en Anthropologie.

[18] Chudzinski T. (1898). Observations sur les variations musculaires dans les races humaines. Mém. Soc. Anthrop. Paris.

[19] Pales L., Chippeaux C. (1952). Myologie comparative du pied. Bull. Mem. Soc. Anthrop. Paris.

[20] Mori M. (1964). Statistics on the musculature of the Japanese. Okajimas Folia Anat. Jpn..

[21] Chaney D.M., Lee M.S., Khan M.A., Krueger W.A., Mandracchia V.J., Yoho R.M. (1996). Study of ten anatomical variants of the foot and ankle. J. Am. Podiatr. Med. Assoc..

[22] Bonnin M., Tavernier T., Bouysset M. (1997). Split lesions of the peroneus brevis tendon in chronic ankle laxity. Am. J. Sports Med..

[23] Kudoh H., Sakai T., Horiguchi M. (1999). The consistent presence of the human accessory deep peroneal nerve. J. Anat..

[24] DiGiovanni B.F., Fraga C.J., Cohen B.E., Shereff M.J. (2000). Associated injuries found in chronic lateral ankle instability. Foot Ankle Int..

[25] Chepuri N.B., Jacobson J.A., Fessell D.P., Hayes C.W. (2001). Sonographic appearance of the peroneus quartus muscle: Correlation with MR imaging. Appearance in seven patients. Radiology.

[26] Borne J., Fantino O., Besse J.L., Clouet P.L., Tran Minh V.A. (2002). Aspect IRM des variants anatomiques des muscles, tendons et ligaments de la Cheville. J. Radiol..

[27] Zammit J., Singh D. (2003). The peroneus quartus muscle. Anatomy and clinical relevance. J. Bone Jt. Surg..

[28] Saupe N., Mengiardi B., Pfirrmann C.W., Vienne P., Seifert B., Zanetti M. (2007). Anatomic variants associated with peroneal tendon disorders: MR imaging findings in volunteers with asymptomatic ankles. Radiology.

[29] Tubbs R.S., May W.R., Shoja M.M., Loukas M., Salter E.G., Oakes W.J. (2008). Peroneotalocalcaneus muscle. Anat. Sci. Int..

[30] Thomas J.L., Lopez-Ben R., Maddox J. (2009). A preliminary report on intra-sheath peroneal tendon subluxation: A prospective review of 7 patients with ultrasound verification. J. Foot Ankle Surg..

[31] Uğurlu M., Bozkurt M., Demirkale I., Cömert A., Acar H.I., Tekdemir I. (2010). Anatomy of the lateral complex of the ankle joint in relation to peroneal tendons, distal fibula and talus: A cadaveric study. Eklem Hast. Cerrahisi.

[32] Saxena A., Luhadiya A., Ewen B., Goumas C. (2011). Magnetic resonance imaging and incidental findings of lateral ankle pathologic features with asymptomatic ankles. J. Foot Ankle Surg..

[33] Prakash Narayanswamy C., Singh D.K., Rajini T., Venkatiah J., Singh G. (2011). Anatomical variations of peroneal muscles: A cadaver study in an Indian population and a review of the literature. J. Am. Podiatr. Med. Assoc..

[34] Athavale S.A., Gupta V., Kotgirwar S., Singh V. (2012). The peroneus quartus muscle: Clinical correlation with evolutionary importance. Anat. Sci. Int..

[35] Nascimento S.R.R., Costa R.W., Ruiz C.R., Wafae N. (2012). Analysis on the incidence of the fibularis quartus muscle using magnetic resonance imaging. Anat. Res. Int..

[36] Clarkson M.J., Fox J.N., Atsas S., Daney B.T., Dodson S.C., Lambert H.W. (2013). Clinical implications of novel variants of the fibularis (peroneus) quartus muscle inserting onto the cuboid bone: Peroneocuboideus and peroneocalcaneocuboideus. J. Foot Ankle Surg..

[37] Bilgili M.G., Kaynak G., Botanlioğlu H., Basaran S.H., Ercin E., Baca E., Uzun I. (2014). Peroneus quartus: Prevalance and clinical importance. Arch. Orthop. Trauma Surg..

[38] Zhenbo Z., Jin W., Haifeng G., Huanting L., Feng C., Ming L. (2014). Sliding fibular graft repair for the treatment of recurrent peroneal subluxation. Foot Ankle Int..

[39] Hur M.S., Won H.S., Chung I.H. (2015). A new morphological classification for the fibularis quartus muscle. Surg. Radiol. Anat..

[40] Galli M.M., Protzman N.M., Mandelker E.M., Malhotra A.D., Schwartz E., Brigido S.A. (2015). An examination of anatomic variants and incidental peroneal tendon pathologic features: A comprehensive MRI review of asymptomatic lateral ankles. J. Foot Ankle Surg..

[41] Somesh M.S., Murlimanju B.V., Roshan S., Kamble G. (2016). Peroneus quartus in south indian population: A Cadaveric study. Int. J. Anat. Res..

[42] Grace S., Preethi Ramya T., Anjana T.S.R. (2016). An adaptational Change of evolutionary significance in peroneal Tendons: An anomalous peroneus quartus and a Proximally migrated peroneus brevis tendon. Int. J. Anat. Res..

[43] Mustafa A.Y.A.E., Mohammed W.A., Alasmari, Alkushi A.G., Sakran A.M.E.A. (2017). Peroneus quartus muscle: Its incidence and Clinical importance. Int. J. Anat. Res..

[44] Dangintawat P., Apinun J., Huanmanop T., Agthong S., Chentanez V. (2019). Morphometric study of inferior peroneal retinaculum and contents of inferior peroneal tunnel. Folia Morphol..

[45] Ersoz E., Tokgoz N., Kaptan A.Y., Ozturk A.M., Ucar M. (2019). Anatomical variations related to pathological conditions of the peroneal tendon: Evaluation of ankle MRI with a 3D SPACE sequence in symptomatic patients. Skelet. Radiol..

[46] Inchai C., Apivatthakakul T., Sinthubua A., Mahakkanukrauh P. (2021). The prevalence of the accessory peroneal muscle; peroneus quartus and its clinical implications. Int. Med. J..

[47] Cheung Y.Y., Rosenberg Z.S., Ramsinghani R., Beltran J., Jahss M.H. (1997). Peroneus quartus muscle: MR imaging features. Radiology..

[48] Yammine K. (2015). The accessory peroneal (fibular) muscles: Peroneus quartus and peroneus digiti quinti. A systematic review and meta-analysis. Surg. Radiol. Anat..

[49] Kassim M.M., Rosenfeld P. (2012). Tendoscopic debridement of peroneus quartus muscle for chronic lateral ankle pain: A case report. Foot Ankle Int..

[50] Lui T.H., Li H.M. (2019). Endoscopic Resection of Peroneus Quartus. Arthrosc. Tech..

[51] Lui T.H. (2012). Tendoscopic resection of low-lying muscle belly of peroneus brevis or quartus. Foot Ankle Int..

